# Characterization of an Omega-3 Desaturase From *Phytophthora parasitica* and Application for Eicosapentaenoic Acid Production in *Mortierella alpina*

**DOI:** 10.3389/fmicb.2018.01878

**Published:** 2018-08-14

**Authors:** Xin Tang, Haiqin Chen, Tiantian Mei, Chengfeng Ge, Zhennan Gu, Hao Zhang, Yong Q. Chen, Wei Chen

**Affiliations:** ^1^State Key Laboratory of Food Science and Technology, Jiangnan University, Wuxi, China; ^2^School of Food Science and Technology, Jiangnan University, Wuxi, China; ^3^National Engineering Research Center for Functional Food, Jiangnan University, Wuxi, China; ^4^Department of Cancer Biology, Wake Forest School of Medicine, Winston-Salem, NC, United States; ^5^Beijing Innovation Centre of Food Nutrition and Human Health, Beijing Technology and Business University, Beijing, China

**Keywords:** omega-3 desaturase, PPD17, *Mortierella alpina*, EPA, carbon/nitrogen source

## Abstract

Omega-3 long-chain polyunsaturated fatty acids (LC-PUFAs) have important therapeutic and nutritional benefits in humans. In the biosynthesis pathways of these LC-PUFAs, omega-3 desaturase plays a critical role. In this study, we report a new omega-3 desaturase (PPD17) from *Phytophthora parasitica*. This desaturase shares high similarities with the known omega-3 desaturases and was expressed in *Saccharomyces cerevisiae* for the activity and substrate specificity research. The desaturase has a wide omega-6 fatty acid substrate, containing both 18C and 20C fatty acids, and exhibits a strong activity of delta-17 desaturase but a weak activity of delta-15 desaturase. The new desaturase converted the omega-6 arachidonic acid (AA, C20:4) to EPA (an omega-3 LC-PUFA, C20:5) with a substrate conversion rate of 70%. To obtain a high EPA-producing strain, we transformed PPD17 into *Mortierella alpina*, an AA-producing filamentous fungus. The EPA content of the total fatty acids in reconstruction strains reached 31.5% and was followed by the fermentation optimization of the EPA yield of up to 1.9 g/L. This research characterized a new omega-3 desaturase and provides a possibility of industrially producing EPA using *M. alpina*.

## Introduction

Omega-3 long-chain polyunsaturated fatty acids (LC-PUFAs), particularly eicosapentaenoic acid (EPA, C20:5) and docosahexaenoic acid (DHA, C22:6), are critical for human health (Xue et al., 2013a). Mammals cannot *de novo* synthesize EPA and DHA, and have to acquire by diet. However, marine microorganisms and phytoplankton can *de novo* synthesize EPA and DHA, and these fatty acids are accumulated in marine fishes through the food chain (Xie et al., 2017). Natural marine fish oil is typically the EPA and DHA source for human consumption, but due to human population growth and oceanic pollution, the current practice of harvesting fish for EPA and DHA is unsustainable (Ji et al., 2015; Okuda et al., 2015).

Many plants, algae and fungi have been investigated as potential hosts to facilitate sustainable commercial EPA or DHA production (Xue et al., 2013a; Sun et al., 2014; Ruiz-Lopez et al., 2015). DHA has been commercially produced in large-scale fermentation processes by using the microalgae (Kyle, 2001), but for a long time, EPA production has not achieved sufficiently high yields. Recently, however, DuPont assembled 30 copies of 9 different heterologous genes in *Yarrowia lipolytica* and the recombination strain accumulated 30% (w/w) lipids in biomass and EPA accounted for 56.6% (w/w) of the total fatty acids (TFAs; Xue et al., 2013b). In contrast to the reconstitution of the EPA biosynthetic pathway in *Y. lipolytica* through the co-expression of a large number of heterologous genes, single-gene modification for EPA production in the microorganisms containing omega-3 LC-PUFA pathways could be simpler and more stable.

*Mortierella alpina*, an oleaginous filamentous fungus producing commercial arachidonic acid (AA, C20:4) used as an additive in infant formulas, has been recognized as a potential strain to commercially produce EPA from AA via an omega-3 desaturase (Sakuradani et al., 2013; Ji et al., 2014). There has been researcher overexpressed an endogenous omega-3 desaturase in *M. alpina* 1S-4, resulting in an increase in the EPA proportion in the TFA content and the maximum value achieved was up to 40% (w/w). However, it had to be grown at a low temperature (12°C) (Ando et al., 2009). Our previous study investigated the omega-3 fatty acid desaturase of *M. alpina* ATCC 32222 and homologously expressed it in *M. alpina*, but the content of EPA was no more than 5% in the TFA. The little EPA production could be attributed to the low activity of the endogenous omega-3 desaturase gene at room temperature. Thus, we need to use high-activity heterologous omega-3 desaturase.

Considerable effort has been made to investigate different omega-3 desaturases from a variety of sources. Researches had shown that the omega-3 desaturases from plants (e.g., *Sapium sebiferum* and *Helianthus annuus*) exclusively desaturated 18C omega-6 PUFAs, and that from *Caenorhabditis elegans* preferentially desaturated 18C PUFAs compared with the 20C substrates (Meesapyodsuk et al., 2000; Venegas-Caleron et al., 2006; Niu et al., 2008). However, some omega-3 desaturases with high activity of delta-17 desaturase in preference to C20 substrates have been discovered recently. The omega-3 desaturase (SDD17) from *Saprolegnia diclina* was found to exclusively desaturate 20C PUFAs with a remarkable preference for AA (Pereira et al., 2004; Okuda et al., 2015). Another omega-3 desaturase (OPIN17) from *Phytophthora infestans* also could convert 31% AA into EPA (Fu et al., 2013). In addition, Xue et al. (2013a) found that three omega-3 desaturases (PaD17 from *Pythium aphanidermatum*, PsD17 from *Phytophthora sojae*, PrD17 from *Phytophthora ramorum*) had strong delta-17 desaturase activity and PaD17 had the highest conversion rate from AA to EPA. Indeed, we had transformed the PaD17 into *M. alpina* and increased the conversion rate of AA to EPA (49.7%) in previous study (Ge et al., 2017).

For commercial EPA production by *M. alpina*, a high-efficiency, ordinary-temperature, and preferential AA substrate omega-3 desaturase is very important. Thus, in this study, we used sequence comparison and structure prediction to study desaturases and found a new omega-3 desaturase defined as PPD17 preferentially converting AA to EPA from *Phytophthora parasitica*. Then, we characterized this new omega-3 desaturase by expressing it in *S. cerevisiae* and transformed it into *M. alpina*. Furthermore, we optimized the culture medium and the conditions of EPA-producing *M. alpina* transformation.

## Materials and Methods

### Sequence Comparison and Structure Prediction

Sequence homology was analyzed by the BLAST tools at NCBI. Sequence alignment was done with the Clustal W software. Initial prediction of the transmembrane (TM) domain was performed through the programs TMHMM, HMMTOP, and TOP-PRED.

### Strains and Plasmid

*Mortierella alpina* CCFM 501 was uracil-auxotrophic through being modified from *M. alpina* ATCC 32222 (Hao et al., 2014). *Agrobacterium tumefaciens* C58C1, which was provided by Yasuyuki Kubo, is a transfer DNA donor for *M. alpina* transformation. *Escherichia coli* Top 10 was used to construct the plasmid. Both the INVSc1 yeast strain and the pYES2/NT C plasmid were purchased from Invitrogen, but the pBIG2-ura5s-ITs plasmid was constructed in our laboratory (Hao et al., 2014).

### Media and Culture

*Escherichia coli* Top 10 was cultivated at 37°C on LB medium and *S. cerevisiae* was grown on YPD medium at 28°C. *M. alpina* strains were grown on the GY medium and 0.05 g/L uracil were added when culturing uracil auxotroph, and *A. tumefaciens* C58C1 was cultured in a YEP medium (Hao et al., 2014). SC-U was the synthetic minimal medium for yeast to induce the recombinant protein expression; its composition is described in our previous work (Shi et al., 2015). The transformation media, including SC medium, MM and IM, were as previously described (Takeno et al., 2004; Ando et al., 2009). When analysing the fatty acid of the *M. alpina* transformants, this fungus was grown for 7 days at 28°C in a modified broth medium (50 g/L glucose, 10 g/L KNO_3_, 5 g/L yeast extract, 1 g/L KH_2_PO_4_⋅H_2_O, 0.25 g/L MgSO_4_⋅7H_2_O).

### Yeast Transformation

The sequence of *PPD17* gene was optimized on the basis of the codon preference of *S. cerevisiae*, and the codon-optimized gene, called *oPPD17*, was synthesized and cloned into the pUCsimple vector. The primer pair *oPPD17* F/*oPPD17* R (**Supplementary Table 1**) was used to amplify *oPPD17*, and the amplification products were ligated into pYES2/NT C to obtain the plasmid pYES2/NT C-*oPPD17*. Then, the obtained plasmid was transformed into INVSc1 through lithium acetate transformation method (Gietz et al., 1992).

### Omega-6 Fatty Acid Feeding

INVSc1 containing pYES2/NT C-*oPPD17* were cultivated on SC-U medium for 48 h and diluted to OD_600_ of 0.4, followed by aliquoting into 20 mL cultures containing 0.2 mM linoleic acid (LA, C18:2), 0.2 mM gamma-linolenic acid (GLA, C18:3), 0.2 mM dihomo-gamma-linolenic acid (DGLA, C20:3) and 0.2 mM AA. When grown for another 48 h, the cultures were collected and the pellets were used for further analysis.

### T-DNA Binary Vector Construction and *Agrobacterium tumefaciens*-Mediated Transformation (ATMT)

The *PPD17* gene was codon-optimized on the basis of the codon preference of *M. alpina*, called *oPpFADS17* (GenBank accession No. KT372001) and then, synthesized and cloned into the pUCsimple vector. The primer pair *oPpFADS17* F/*oPpFADS17* R (**Supplementary Table 1**) was used to amplify *oPpFADS17*, and the *oPpFADS17* gene was cloned into pBIG2-ura5s-Its to acquire pBIG2-ura5s-*oPpFADS17*. The vector carrying the *oPpFADS17* gene was used for ATMT as previously described (Hao et al., 2014).

### RT-qPCR Analysis

The total RNA of the transformants was isolated with TRIzol (Invitrogen, United States) and then converted to cDNA by the PrimeScript RT reagent kit (Takara, Japan). The quantitative PCR was carried out through CFX Connect RealTime System (Bio-Rad, United States). The PCR amplification involved 50°C for 2 min, 95°C for 10 min, followed by 40 cycles of 95°C for 15 s and 58°C for 30 s. The primer pairs are showed in **Supplementary Table 1**, and the 18S rRNA is the internal control in the PCR amplification. The relative transcription levels were calculated using the 2^-ΔΔC_t_^ method (Shi et al., 2016). The introduced gene is exogenous and it is absent in control strains, thus one of the transformants was selected as standard for relative transcription level analysis.

### Western Blot Analysis

Western blot analysis was carried out as described in our previous study (Chen et al., 2013). Briefly, 100 μg of cellular protein was loaded onto SDS-PAGE (12% gel), and PVDF membranes (Millipore, United States) were used for the analysis. The Anti-His antibodies (Nanjing GenScript Biotechnology Corporation, China) were used to detect the recombinant protein.

### Cell Dry Weight (CDW) and Fatty Acid Methyl Ester (FAME) Analysis

The biomass was collected through filtration and washed, followed by being freeze-dried; then, CDW was determined gravimetrically. Extraction and methyl esterification of TFAs were carried out as described previously (Wang et al., 2011). Fatty acid methyl ester (FAME) analysis was used GCMS-QP2010 Ultra (Shimadzu Co., Japan) and the temperature program was described in our previous work (Hao et al., 2016).

## Results

### Bioinformatics Analysis of the New Sequence PPD17 From *Phytophthora parasitica*

**FIGURE 1:**
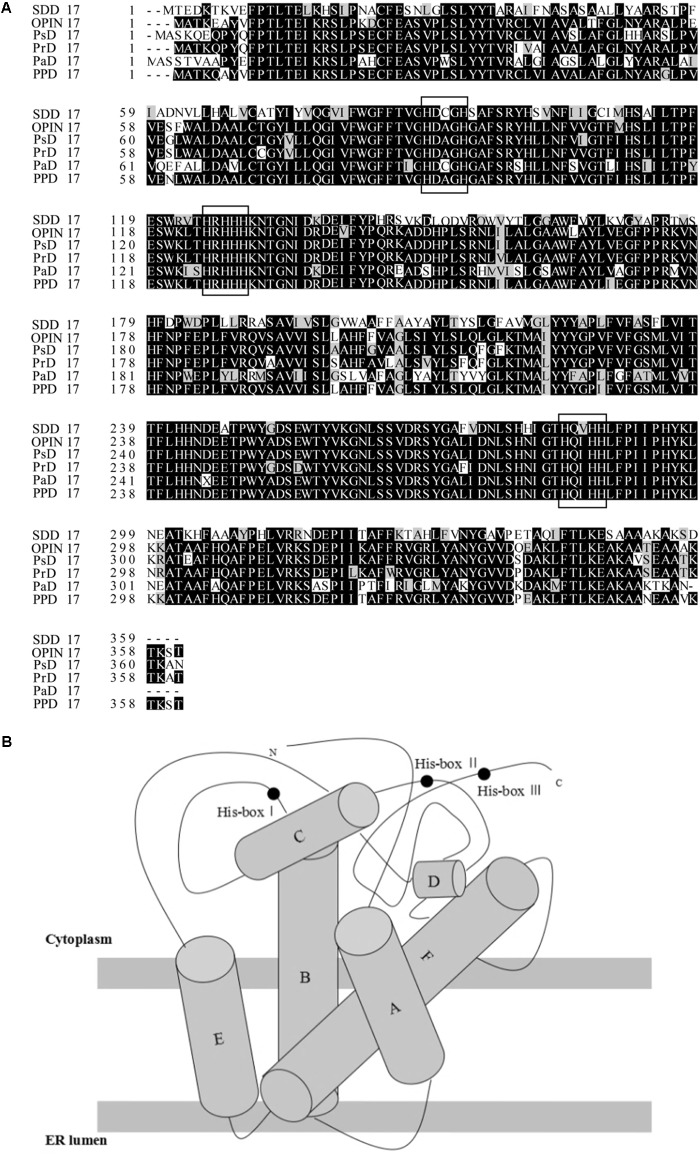
**(A)** Sequence comparison of selected omega-3 desaturases using the Clustal W (SDD17, *Saprolegnia diclina* delta-17 desaturase; OPIN17, *Phytophthora infestans* delta-17 desaturase; PrD17, *Phytophthora ramorum* delta-17 desaturase; PsD17, *Phytophthora sojae* delta-17 desaturase; PaD17, *Pythium aphanidermatum* delta-17 desaturase; PPD17, a sequence from *Phytophthora parasitica*). **(B)** Topology model of omega-3 desaturase PPD17.

The sequence named PPD17 from *P. parasitica* searched from GenBank which is 1,086 bp and encodes a peptide of 361 amino acids was aligned with several known omega-3 desaturases using Clustal W (**Figure 1A**). The sequence shares high similarities (>60%) with known desaturases. Conserved motifs were identified and all of these conserved motifs included the three histidine boxes that are known to be required for desaturase activity. The structure arrangements of all these desaturases are very similar and consist of four membrane-spanning domains and one catalytic domain on the cytosolic side of the membrane (**Figure 1B**). The predicted topology model was in accordance with other membrane-bound enzymes. The protein also contains KTKST, which was the C terminal motif, proposing to be a retention signal for a number of TM proteins in ER (endoplasmic reticulum).

### Characterization of Substrate Specificity of PPD17 in *S. cerevisiae*

**FIGURE 2:**
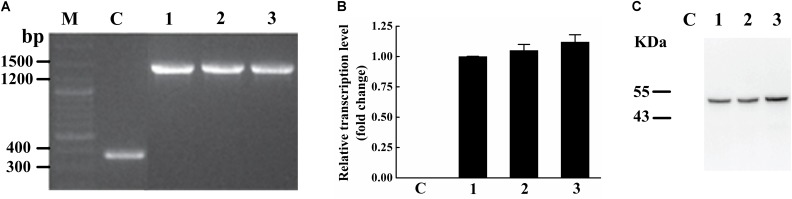
Verification of transformants expressing PPD17. **(A)** Map of fragment bands in the genome. **(B)** PPD17 relative transcription level. **(C)** PPD17 translation level. M, nucleic acid marker; C, INVSc1-pYES2/NT control strains; 1–3, INVSc1 (pYES2/NT C-*oPPD17-1/2/3*).

**FIGURE 3:**
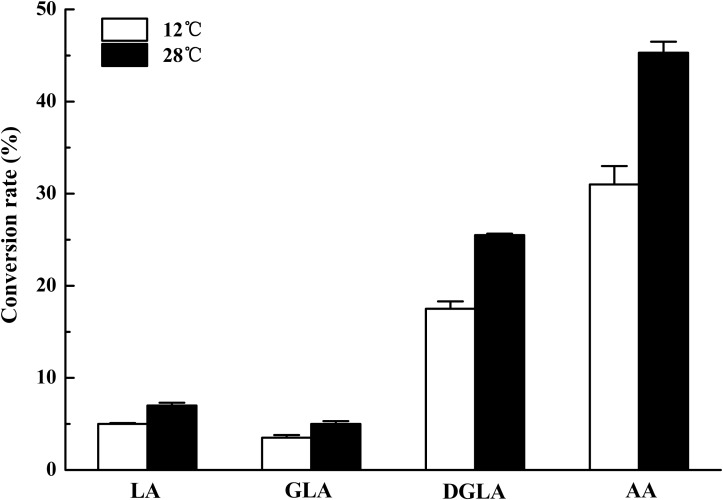
Conversion rate of yeast transformants on different fatty acids at different temperature. The transformants were cultivated in 0.2 mM LA, GLA, DGLA, and AA. Conversion rate is calculated as 100% × Product/(Product + Substrate).

**FIGURE 4:**
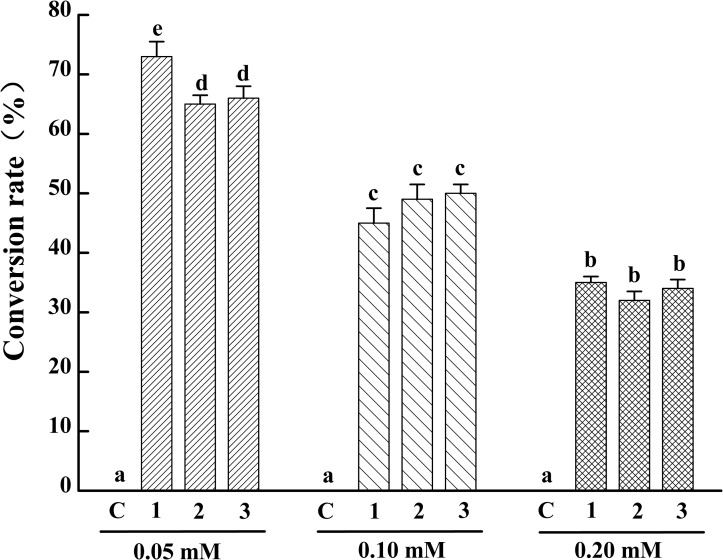
Conversion rate of yeast transformants with different concentration AA adding. C, Control, INVSc1 (pYES2/NT C); 1–3, yeast transformants harboring pYES2/NT C-*oPPD17*-1/2/3. Values share no common superscripts were significantly different (*P* < 0.05).

To identify the activity and characterize the substrate specificity of PPD17, the desaturase was codon optimized and expressed in *S. cerevisiae*. The results of DNA agarose electrophoresis, RT-qPCR and Weston bolt analysis showed that oPPD17 was successfully expressed, transcribed and translated in the recombinant strains INVSc1 (**Figure 2**). To acquire insight into substrate requirements of PPD17, different fatty acid substrates were supplied in the media. The transformant INVSc1 containing pYES2/NT C-*oPPD17* grown on the media supplemented with LA (converting into α-linolenic acid, ALA, C18:3), GLA (converting into stearidonic acid, SDA, C18:4), DGLA (converting into eicosatetraenoic acid, ETA, C20:4) and AA (converting into EPA), respectively, at 12°C°C and 28°C (**Figure 3**). The results indicated PPD17 convert 18C-PUFAs at a low efficiency (<10%) while the efficiency on 20C-PUFAs is much higher, especially AA, which almost reached up to 50%. In addition, compared with low temperature (12°C), the conversion rate was high at ordinary temperature (28°C), which was beneficial for the application of this enzyme. Furthermore, the effects of different AA-added concentration on the conversion rate of the yeast transformants was analyzed, it was found that AA conversion rate decreased as AA-added concentration increasing and the highest conversion rate (∼70%) was achieved when added 0.05 mM AA (**Figure 4**).

### Transformation of PPD17 in *M. alpina* and Fatty Acid Analysis

**FIGURE 5:**
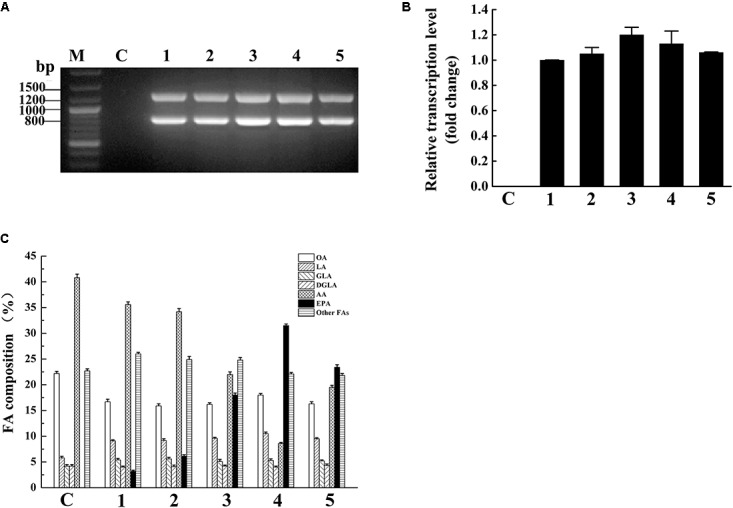
Verification of oPpFADS17 fragments in the genome **(A)**, and relative transcription level **(B)**, and fatty acid composition **(C)** in *M. alpina* expressing oPpFADS17. M, marker; C, *M. alpina* (control); 1–5, *M. alpina*-*oPpFADS17*-(1–5).

*Mortierella alpina* can accumulated a large number of AA (more than 50% of total fatty acids) (Wang et al., 2011) and PPD17 has a great capability of converting AA to EPA, therefore we codon optimized PPD17 gene (*oPaFADS17*) and heterologously expressed it in *M. alpina* auxotrophic strain for EPA production. In this study, five stable transformants were acquired. The *ura5* (818 bp) and *oPpFADS17* (1,250 bp) fragments indicate that the *oPpFADS17* were successful inserted into the *M. alpina* transformants’ genome (**Figure 5A**). The RT-qPCR analysis showed that *oPpFADS17* was successfully transcribed in all of the five transformants (**Figure 5B**).

Fatty acid methyl ester analysis showed that the transformants and wild-type *M. alpina* had a similar fatty acid composition except for AA and EPA (**Figure 5C**). The wild-type *M. alpina* had no significant EPA accumulation. Although all of the five transformants could accumulate EPA, their EPA accumulation capacity were obviously different due to random integration based on ATMT method (Ando et al., 2009), which resulting in different effect on EPA production. Among all of the transformants, MA-*oPpFADS17*-4 (named *M. alpina* CCFM 698) exhibited the strongest capacity to accumulate EPA, and the EPA content in the TFA increased up to 31.5%. In this transformant, the accumulated AA decreased to 8.6% and the conversion rate of AA to EPA reached 78.6%.

### Effects of Carbon/Nitrogen Source on EPA Production

**FIGURE 6:**
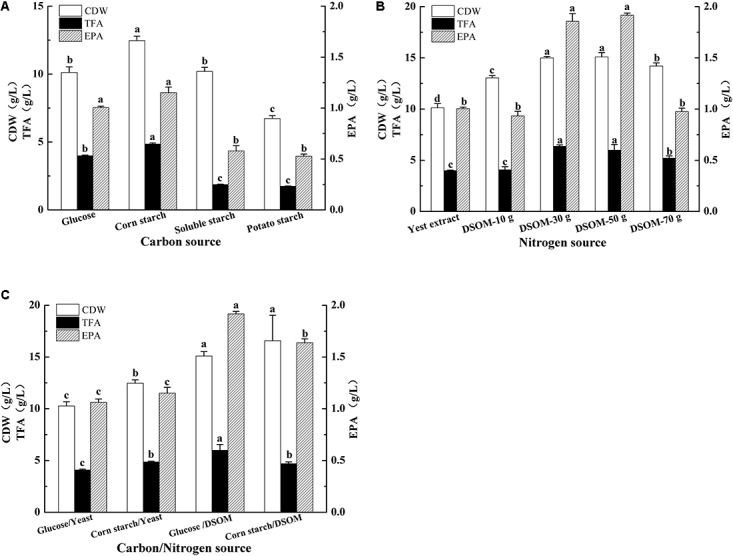
Effects of different carbon **(A)**, nitrogen **(B)**, combination **(C)** sources on cell dry weight (CDW), total fatty acids (TFAs), and EPA production of the recombinant *M. alpina* CCFM 698.

The carbon substrates are important to cell growth and lipid accumulation in oleaginous microorganisms, and *M. alpina* can use a variety of inexpensive starch-based carbon sources for lipid production (Zhu et al., 2003; Nisha and Venkateswaran, 2011). To enhance EPA production and reduce the fermentation cost, the effects of different carbon sources on CDW, TFA, and EPA production in the recombinant *M. alpina* CCFM 698 were investigated (**Figure 6A**). Compared to other carbon sources, corn starch stimulated the growth of this fungus and the CDW reached up to 12.5 g/L, which was 24% high than that of glucose (10.1 g/L). However, the CDW grown on soluble starch and potato starch were no more than glucose. The TFA and EPA yield revealed corn starch and glucose are beneficial to TFA and EPA accumulation. The EPA production grown on corn starch and glucose were up to 1.2 g/L and 1.0 g/L, respectively, which were significantly higher than that grown on soluble starch and potato starch. Thus, corn starch and glucose are favorable carbon sources for EPA production in *M. alpina* CCFM 698.

Nitrogen are key components in the growth media for microorganisms and the inexpensive nitrogen source soybean meal had been reported to enhance the biomass and fatty acid production in *M. alpina* (Cao et al., 2015). As the mycelia and soybean meal are hard to be separated during biomass collection, *M. alpina* CCFM 698 was grown on defatted soybean meal (DSOM) in this study. As shown in **Figure 6B**, compared to yeast extract, the high EPA-produced strain grown on 30 g/L and 50 g/L DSOM achieved higher CDW, TFA, and EPA yield. When *M. alpina* CCFM 698 was grown on 50 g/L DSOM, the EPA yield reach the highest value 1.9 g/L.

These above results suggested corn starch and glucose are favorable carbon sources, and 50 g/L DSOM is appropriate nitrogen source for EPA production in the high EPA-produced strain *M. alpina* CCFM 698. To obtain the optimal carbon/nitrogen source recipe, this strain was grown on glucose/DMSO and corn starch/DMSO, respectively, and using the yeast extract nitrogen source as control (**Figure 6C**). Compared with corn starch/DSOM, glucose/DMSO gave higher TFA and EPA yield although their CDW had no significant difference, this may due to the glucose is more beneficial to lipid accumulation. Thus, in this study, the highest EPA production was obtained when grown in glucose and DSOM, and the highest EPA yield was up to 1.9 g/L.

## Discussion

*Phytophthora* is known to produce LC-PUFAs (e.g., AA and EPA), and synthesize these PUFAs via desaturase/elongase pathway rather than polyketide pathway (Fu et al., 2013; Xue et al., 2013a). Thus, it is highly possible to screen new desaturase or elongase genes for EPA pathway in these microorganisms. In this study, a new omega-3 desaturase in *P. parasitica* was identified and named PPD17. The new sequence PPD17 has high similarities with the published desaturases (Pereira et al., 2004; Fu et al., 2013; Xue et al., 2013a; Okuda et al., 2015), and contains three typical histidine motifs HDAGH, HRHHH, HQIHH, suggesting it owns the characteristic of membrane-bound desaturase. These motifs were proposed to be related to the iron atoms ligation in the active-site. Meanwhile, PPD17 contains four high hydrophobic regions clusters, which are the putative membrane spanning helices.

Omega-3 desaturase (e.g., delta-17 desaturase) is necessary for conversion of omega-6 PUFAs into their omega-3 counterparts and play a vital role in EPA synthesis from AA (Xue et al., 2013a; Xie et al., 2015). The activity and substrate preference of PPD17 are analyzed by expressing the protein in *S. cerevisiae*. Fatty acid feeding experiments showed that PPD17 had stronger substrate preference for C20-PUFA substrate AA and DGLA, especially AA, and lower preference for C18-PUFA substrate LA and GLA, which was in according with the identified delta-17 desaturase PaD17, PrD17, and PsD17 (Xue et al., 2013a). However, the other delta-17 desaturase SDD17 was found to be an exclusive desaturase for C20-PUFA and had no activity on C18 substrates (Pereira et al., 2004). In contrast to SSD17, the PPD17 had the activity of delta-15 desaturase and can convert LA and GLA to ALA and SDA, respectively. When LA and AA were fed simultaneously, the conversation rate of AA was much higher than LA, further indicating that PPD17 is much more preference AA. The highest conversion rate to AA can reach 70% when 0.05 mM AA was added at ordinary temperature 28°C, suggesting that PPD17 has great potential to produce EPA from AA.

Oleaginous fungus *M. alpina* can accumulate 50% AA of total fatty acid composed by different 18C- and 20C-PUFAs (Wang et al., 2011). Furthermore, *M. alpina* also can accumulate little EPA at low temperature (Shimizu et al., 1989; Sakuradani et al., 2005). There has been researcher attempted to enhance EPA production in *M. alpina* through overexpressing endogenous omega-3 desaturase, but the EPA production was no more than 0.7 g/L and needed to be cultured at 12°C rather than ordinary temperature (Takeno et al., 2004). For engineering an organism with high EPA production, the conversion efficiency of the omega-3 desaturase used is one of the important factors, and the high activity of the newly identified PPD17 render its excellent choices for genetic engineering in the EPA production. In the present study, the codon optimized PPD17 gene was successful heterologously expressed in *M. alpina* using the *A. tumefaciens* transformation method and the conversion rate of AA to EPA in a transformant MA-*oPpFADS17*-4 reached up to 78.6%, which were higher than that in engineered EPA-produced *Y. lipolytica* strains which harbored the omega-3 desaturase PaD17, PrD17, or PsD17 (Xue et al., 2013a). Furthermore, the conversion rate of AA to EPA of PPD17 in this strain was also much higher than PaD17 (49.7%) from our previous study (Ge et al., 2017). The EPA content of the TFA in PPD17-harboring *M. alpina* transformant increased up to 31.5%, which was higher than PaD17-harboring *M. alpina* transformant (18.7%) (Ge et al., 2017), but less than that of recombination *Y. lipolytica* (56.6%) which assembled 30 copies of nine different heterologous genes (Xue et al., 2013b; Xie et al., 2015). However, *M. alpina*, as an oleaginous filamentous fungus producing commercial AA, was introduced a single-copy of PPD17 coding gene and was able to convert nearly 80% AA to EPA. *M. alpina* transformant integrated PPD17 may have great potential for future commercial EPA production.

Carbon substrates and nitrogen sources are very vital chemical compounds in microorganism cultivation. *M. alpina* can use various carbon and nitrogen sources for growth and lipid accumulation (Nisha and Venkateswaran, 2011; Yu et al., 2011). Our results demonstrated the inexpensive carbon source corn starch enhanced the biomass and TFA production in the recombinant *M. alpina* CCFM 698. For the development of PUFAs production by large-scale fermentation process, it is necessary to utilize inexpensive medium components to reduce the cost (Zhu et al., 2003). Thus, the corn starch has a potential of industrialize applications as carbon source for EPA production in *M. alpina*. For nitrogen source, organic nitrogen compounds are in favor of cell growth and lipid accumulation compared with inorganic nitrogen sources (Lu et al., 2011). Compared with the common organic nitrogen yeast extract, the DSOM obviously increased the biomass, TFA, and EPA yield in *M. alpina* CCFM 698, which is in accordance with the recent finding that the inexpensive nitrogen source soybean meal could increase the biomass and fatty acid production in *M. alpina* (Cao et al., 2015). Indeed, the highest EPA yield reached up to 1.9 g/L when the recombinant grown on 50 g/L DSOM at ordinary temperature in this study. This EPA yield was much higher than that (0.6 g/L) in our previous engineered strain which was heterogeneous expressed an ALA-preferring delta-6 desaturase (Shi et al., 2016). Nevertheless, there has been other study reported that EPA production was up to 1.8 g/L through expressing the *S. diclina* delta-17 desaturase, which was little less than our result (Okuda et al., 2015). Furthermore, the EPA yield of PPD17-harboring *M. alpina* transformant in this study was also higher than PaD17-harboring transformant which yield 1.7 g/L EPA after a series of fermentation optimization (Ge et al., 2017). PPD17 was first discovered and characterized in this study and the conversion rate of AA to EPA with PPD17 was much higher than PaD17.

In general, this recombinant *M. alpina* CCFM 698 has great potential for EPA industrialized production. Furthermore, although optimal carbon/nitrogen source recipe indicated that the glucose/DMSO gave higher EPA yield than corn starch/DSOM, the low cost of corn starch might make this carbon source own a broad application prospect.

## Conclusion

In conclusion, a new omega-3 desaturase PPD17 from *Phytophthora parasitica* was reported in this study. This new omega-3 desaturase shares high similarities with known omega-3 desaturases and has a wide omega-6 fatty acid substrate preference including both 18-C and 20-C omega-6 fatty acids with strong delta-17 desaturase activity but weaker delta-15 desaturase activity. The conversion rate from AA to EPA was up to 70% when this desaturase was expressed in *S cerevisiae*. For obtaining a high EPA-producing strain, this desaturase was transformed into *Mortierella alpina*, an AA-producing filamentous fungus. The EPA content of total fatty acid in reconstruction strains reached 31.5% and followed by fermentation optimization the EPA yield was up to 1.9 g/L.

## Author Contributions

XT, TM, and CG carried out the experiments and drafted the manuscript. HC and ZG analyzed the data and helped to draft the manuscript. HC, HZ, YC, and WC conceived and designed the study and revised the manuscript. All authors read and approved the final manuscript.

## Conflict of Interest Statement

The authors declare that the research was conducted in the absence of any commercial or financial relationships that could be construed as a potential conflict of interest. The reviewer GP and handling Editor declared their shared affiliation at time of review.
