# Supplementary material for: Characterization of an Omega-3 Desaturase From Phytophthora parasitica and Application for Eicosapentaenoic Acid Production in Mortierella alpina
Source: Front Microbiol. 2018 Aug 14;9:1878. doi: 10.3389/fmicb.2018.01878 (PMC6102326; doi:10.3389/fmicb.2018.01878)
Supplement: Supplementary file 1 [file Table_1.DOCX]

**Characterisation of an omega-3 desaturase from *Phytophthora parasitica* and application for eicosapentaenoic acid production in *Mortierella alpina***

Xin Tang ^1, 2^, Haiqin Chen ^1, 2,^ *, Tiantian Mei ^1, 2^, Chengfeng Ge ^1, 2^, Zhennan Gu ^1, 2^, Hao Zhang ^1, 2^, Yong Q. Chen ^1, 2, 3, 4^, Wei Chen ^1, 2, 3, 5^

^1^ State Key Laboratory of Food Science and Technology, Jiangnan University, Wuxi, Jiangsu, China

^2^ School of Food Science and Technology, Jiangnan University, Wuxi, Jiangsu, China

^3^ National Engineering Research Center for Functional Food, Jiangnan University, Wuxi, Jiangsu, China

^4^ Department of Cancer Biology, Wake Forest School of Medicine, Winston-Salem, NC, USA

^5^ Beijing Innovation Centre of Food Nutrition and Human Health, Beijing Technology and Business University, Beijing, China

*Corresponding author.

E-mail address: [haiqinchen@jiangnan.edu.cn](mailto:haiqinchen@jiangnan.edu.cn) (Haiqin Chen)

Telephone： +86 510 85197239

Fax: +86 510 85197096

**Supplementary material**

**Table 1 Primers used in this study**

| Primer name | Restriction enzyme | Oligonucleotide sequence (5'-3')^a^ | Function |
| --- | --- | --- | --- |
| ***Saccharomyces cerevisiae*** | | | |
| *oPPD17-*F | *EcoR* I | CTAATTGAATTCATGGCAACCAAGCAAGC | *oPPD17* amplification for plasmid construction |
| *oPPD17-*R | *Xho* I | CGATTCTCGAGTTAAGTTGACTTGGTTTTAACAGCG |  |
| q- *oPPD17-*F | *-* | GGCAACCAAGCAAGCCTATGTA | qPCR for *oPPD17* transcription level measurement |
| q- *oPPD17-*R | *-* | GCTAAGGCAACTGCAATTACCAAAC |  |
| 18S F | - | AATCATCAAAGAGTCCGAAGACATTG | the internal control gene for qPCR |
| 18S R | - | CCTTTACTACATGGTATAACTGTGG |  |
| ***Mortierella alpina*** | | | |
| *oPpFADS17-*F | *Hin*d III | ATCTTGAAGCTTCAATGGCTACCAAGCAGGCC | *oPpFADS17* amplification for plasmid construction |
| *oPpFADS17*-R | *Xho* I - | TCTAGACTCGAGTTAGGTGGACTTGGTCTTGACAG |  |
| q-*oPpFADS17*-F | - | TCTTCCCCACCCTCACCG | qPCR for *oPpFADS17* transcription level measurement |
| q-*oPpFADS17-*R | - | CAAGCCACGAGCGTAGTTCA |  |
| 18SRTF | - | CGTACTACCGATTGAATGGCTTAG | the internal control gene for qPCR |
| 18SRTR | - | CCTACGGAAACCTTGTTACGACT |  |
| HisproF | - | CACACACAAACCTCTCTCCCACT | T-DNA insert detection for binary plasmid construction |
| TrpCR | - | CAAATGAACGTATCTTATCGAGATCC |  |

^a^ Underlined sequences indicate the additional restriction sites.
